# Listening to Mom in the NICU: effects of increased maternal speech exposure on language outcomes and white matter development in infants born very preterm

**DOI:** 10.1186/s13063-021-05385-4

**Published:** 2021-07-13

**Authors:** Edith Brignoni-Pérez, Maya Chan Morales, Virginia A. Marchman, Melissa Scala, Heidi M. Feldman, Kristen Yeom, Katherine E. Travis

**Affiliations:** 1 Division of Developmental-Behavioral Pediatrics, Department of Pediatrics, Stanford University School of Medicine, Stanford, CA USA; 2 Department of Psychiatry and Behavioral Sciences, Stanford University School of Medicine, Stanford, CA USA; 3 Department of Psychology, Stanford University, Stanford, CA USA; 4 Division of Neonatology, Department of Pediatrics, Stanford University School of Medicine, Stanford, CA USA; 5 Department of Radiology, Stanford University School of Medicine, Stanford, CA USA

**Keywords:** Preterm, Language, Brain, NICU, Intervention, Diffusion MRI, White matter, Clinical trial

## Abstract

**Background:**

Infants born very preterm (< 32 weeks gestational age (GA)) are at risk for developmental language delays. Poor language outcomes in children born preterm have been linked to neurobiological factors, including impaired development of the brain’s structural connectivity (white matter), and environmental factors, including decreased exposure to maternal speech in the neonatal intensive care unit (NICU). Interventions that enhance preterm infants’ exposure to maternal speech show promise as potential strategies for improving short-term health outcomes. Intervention studies have yet to establish whether increased exposure to maternal speech in the NICU offers benefits beyond the newborn period for brain and language outcomes.

**Methods:**

This randomized controlled trial assesses the long-term effects of increased maternal speech exposure on structural connectivity at 12 months of age (age adjusted for prematurity (AA)) and language outcomes between 12 and 18 months of age AA. Study participants (*N* = 42) will include infants born very preterm (24–31 weeks 6/7 days GA). Newborns are randomly assigned to the treatment (*n* = 21) or standard medical care (*n* = 21) group. Treatment consists of increased maternal speech exposure, accomplished by playing audio recordings of each baby’s own mother reading a children’s book via an iPod placed in their crib/incubator. Infants in the control group have the identical iPod setup but are not played recordings. The primary outcome will be measures of expressive and receptive language skills, obtained from a parent questionnaire collected at 12–18 months AA. The secondary outcome will be measures of white matter development, including the mean diffusivity and fractional anisotropy derived from diffusion magnetic resonance imaging scans performed at around 36 weeks postmenstrual age during the infants’ routine brain imaging session before hospital discharge and 12 months AA.

**Discussion:**

The proposed study is expected to establish the potential impact of increased maternal speech exposure on long-term language outcomes and white matter development in infants born very preterm. If successful, the findings of this study may help to guide NICU clinical practice for promoting language and brain development. This clinical trial has the potential to advance theoretical understanding of how early language exposure directly changes brain structure for later language learning.

**Trial registration:**

NIH Clinical Trials (ClinicalTrials.gov) NCT04193579. Retrospectively registered on 10 December 2019.

## Background

Each year, approximately 400,000 infants in the United StatesUSA and 15 million worldwide are born preterm (before 37 weeks of gestation) [1]. Up to 50% of infants born less than 32 weeks of gestation develop disadvantaged outcomes, including language and related learning difficulties [2–7]. Poor language skills can lead to poor social relationships [8], academic and occupational underachievement [9], and high utilization of special education [6]. Although many studies of premature birth describe poor language outcomes [2, 6, 7, 10, 11], relatively few propose interventions beyond medical treatment [12].

Poor language outcomes in preterm children have been attributed in part to the minimal amount of maternal speech that neonates experience while hospitalized in the neonatal intensive care unit (NICU) [12–14]. Under typical developmental circumstances, maternal speech is one of the most salient acoustic stimuli experienced by a fetus [15, 16]. The sound environment of an open-bay NICU has been estimated to contain only 2–5% (~ 50 min/day) of adult speech sounds [17]. Studies in older, typically developing infants have demonstrated that the quantity and quality of language input a child experiences are tightly linked to later language skills [18, 19]. Maternal speech input during the first postnatal year is also shown to assist infants’ abilities to recognize speech sounds [20–22]. One observational study of preterm newborns found that variations in the amount of adult speech a newborn heard on a single day in the NICU at 32 and 36 weeks postmenstrual age (PMA) were positively associated with language skills at 7 and 18 months [17]. Small interventional studies have found that experimentally increasing preterm infants’ exposure to maternal speech can significantly improve short-term health outcomes [14], including improvements in oxygen saturation [23, 24], decreases in apnea and bradycardia events [25, 26], improvements in weight gain [26], feeding tolerance [27, 28], and auditory cortex thickness as measured by cranial ultrasound [29]. It is not known yet, however, whether there are sustained long-term benefits of early maternal speech exposure on language outcomes.

Adverse neurodevelopmental and language outcomes in preterm children have also been attributed in part to the susceptibility of white matter to damage from oxidative stress induced by common complications of preterm birth, such as hypoxia, ischemia, and inflammation [30]. Although advances in medical care have reduced the incidences of severe white matter brain injuries (e.g., periventricular leukomalacia), diffuse white matter abnormalities remain a common sequelae of preterm birth. These more subtle white matter abnormalities are more readily detected using diffusion magnetic resonance imaging (dMRI) than with conventional MRI or ultrasound imaging methods. dMRI is an advanced MRI technique used to assess microstructural properties of white matter pathways (or tracts). Metrics derived from dMRI for assessing white matter microstructure include measures such as mean diffusivity (MD) and fractional anisotropy (FA). Studies of preterm newborns have shown these white matter metrics to differ in comparison with term-born infants [31] and to relate to language outcomes at two years of age [32]. In older term-born children, dMRI measures have been found to vary in relation to how much speech children experience from caregivers [33]. Evidence for whether increased speech exposure in the NICU promotes structural changes in white matter connectivity remains limited.

Our aim is to establish whether a language intervention (treatment) administered in the NICU has the potential to promote healthy language and brain development in very preterm infants. To achieve this goal, we have designed an interventional randomized controlled trial (RCT) that will test the causal effect of increased exposure to maternal speech in the NICU on language outcomes and white matter development in infants born very preterm. Our first aim will assess the long-term effects of increased maternal speech exposure on an infant’s expressive and receptive language abilities measured at 12–18 months of age adjusted for prematurity (AA). We focus on these language abilities because they have been linked to language processing abilities that are known to be strong predictors of later academic outcomes [34–36]. We hypothesize that, compared to controls, infants in the treatment group will demonstrate more advanced language skills at 12–18 months AA. Our second aim will assess the effects of increased maternal speech exposure on an infant’s white matter development at ~ 36 weeks PMA and 12 months AA. Measuring white matter development at these two ages will allow us to assess whether possible immediate effects of increased maternal speech exposure on white matter development are sustained over the first year of life. We hypothesize that, compared to controls, infants in the treatment group will demonstrate changes in dMRI measures reflecting increased white matter development. Specifically, infants in the treatment group will demonstrate lower MD.

## Methods

### Study design

**Fig. 1:**
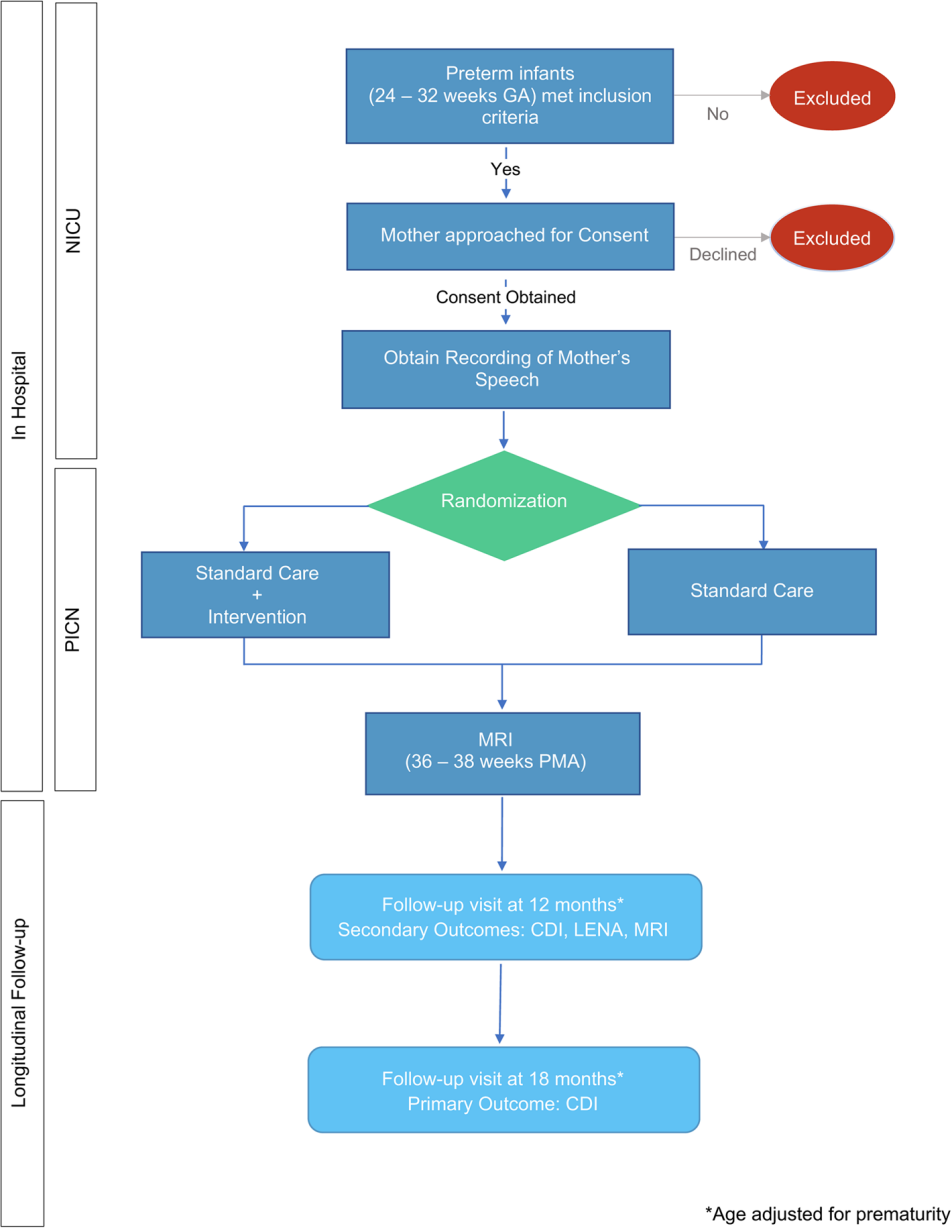
Flow chart of the trial protocol. CDI, MacArthur-Bates Communicative Development Inventories; GA, gestational age; LENA, language environment analysis; MRI, magnetic resonance imaging; NICU, neonatal intensive care unit; PICN, Packard intermediate care nursery; PMA, postmenstrual age

To achieve these aims, we propose an RCT that will involve 42 very preterm infants born and cared for at Stanford’s Lucile Packard Children’s Hospital (LPCH). Enrollment is planned from November 2019 to December 2021. For infants who meet the inclusion criteria of gestational age at birth (GA) of 24-0/7 to 31-6/7 weeks, we will obtain informed consent and questionnaires about the family’s demographics and language from parents, and a speech recording from each mother. Infants will then be randomized to the treatment (T) or control (C) group. Infants randomized to either T or C group will receive standard medical care, with the exception that infants randomized to the T group will also receive the maternal speech intervention achieved by playing recordings of maternal voice via iPods placed in an infant’s incubator or open crib. Infants in the C group will have the same auditory setup to ensure that parents and clinical staff remain blinded to the group status. The duration of the intervention will begin once an infant gets transferred to the intermediate care nursery, indicating medical stability, and end once an infant has received their clinical MRI, which is part of the standard medical care at LPCH and is performed prior to hospital discharge (~ 36–38 weeks PMA). The long-term effects of the intervention will be assessed at 12 months and 18 months AA. At 12 months AA, infant participants will undergo a dMRI scan during natural sleep, and parents will complete the MacArthur-Bates Communicative Development Inventories (CDI): Words and Gestures Questionnaire [37]. At 18 months AA, parents will complete another CDI: Words and Gestures Questionnaire, the primary outcome assessment of language development (see Fig. 1).

### Participants

Preterm neonates born and admitted to the LPCH’s NICU at Stanford University will be eligible to participate. To be eligible, neonates must be born between 24-0/7 and 31-6/7 weeks GA (*N* = 42; T: *n* = 21 and C: *n* = 21). All races and ethnicities will be included. However, we will enroll infants whose family’s primary language is English or Spanish, because of our limitations in communicating with families using other languages. The primary language outcome, the CDI: Words and Gestures Questionnaire, will be administered in English or Spanish, as appropriate [37, 38]. Eligible participants must be free of the following adverse outcomes that could potentially confound language and neuroimaging outcomes: (1) congenital anomalies or recognizable malformation syndromes; (2) serious neurological conditions, including active seizure disorders, history of central nervous system infections or hydrocephalus, intraventricular hemorrhage grades III–IV, and cystic periventricular leukomalacia; (3) surgical treatment for necrotizing enterocolitis; (4) small for GA (< 3 percentile) and/or intra-uterine growth restriction; and (5) major sensori-neural hearing loss.

### Intervention design

#### Enrollment procedures

Families of eligible preterm neonates will be approached for participation and consent when their child is close to moving from the NICU to the step-down unit, called the Packard Intermediate Care Nursery (PICN), indicating that they are medically stable. This procedure also ensures that the auditory capacities of neonates are adequately developed to perceive speech [39]. Moreover, the PICN is generally a quieter environment suitable for playing voice recordings.

#### Speech recording procedures

Language samples will be obtained from each mother as she reads a chapter from a children’s storybook that is available in English and in Spanish. Speech recording will be obtained prior to randomization to mask parents and research staff to group assignment. We will counsel all mothers to imagine reading to the infant. Recordings will be approximately 30 min and normalized for intensity and segmented using an auditory software (Praat: http://www.fon.hum.uva.nl/praat/) [40].

#### Randomization and RCT design elements

Eligible participants will be randomized by the principal investigator (KET), who will not be blinded, in order to provide close monitoring of the procedures. The principal investigator will allocate participants sequentially as they are enrolled to either the T or the C group using the minimization algorithm by Pocock and Simon [41] implemented in the R statistical software package [42]. Randomization will be stratified for (1) GA (24-0/7 to 27-6/7 weeks or 28-0/7 to 31-6/7 weeks), to control for potential development differences in response to the intervention and as a proxy for neural injury, and (2) socioeconomic status (SES) (above versus below average SES; based on Hollingshead Index (HI) [36, 43]), to control for potential differences in language outcomes affected by socioeconomic factors. Taking into account ethical considerations and parental preferences, twins and multiples will be assigned to the same group [44]. Families and research and clinical staff will be blinded to the group status. In the unlikely event that a family, research staff, or clinical staff suspect that an infant may not tolerate the speech recording, the principal investigator will reveal the group assignment.

#### Delivery of intervention

The intervention will occur for a minimum of 2 weeks (28 h) and a maximum of 9 weeks (126 h) prior to the date of the clinical brain imaging scan. Neonates randomized to the T group (maternal speech group) will listen, via an iPod placed in cribs and/or incubators, to recordings of their mother’s speech at hourly intervals between 10:00 p.m. and 6:00 a.m. Administration of the intervention during periods when parents are unlikely to visit the hospital is expected to minimize parental knowledge for the group assignment. Recordings will be played automatically using the alarm function to avoid reliance on clinical staff. Research staff will regularly check on the status of the device to ensure proper functioning. Neonates will hear a total of 2.67 h of speech recordings per night (20 min/h × 8 h). Treatment length will be defined as the number of nights from the start of treatment (i.e., beginning of PICN stay) to the end of treatment (i.e., date of MRI scan at 36–38 weeks PMA). Within a given hour, two 10-min segments will be randomly presented to avoid synchronization with biological and sleep rhythms. Sound intensity for speech recordings is balanced between open cribs versus incubators and is below hourly NICU safety levels less than 50 dB [45].

Neonates in the C group will receive standard care. All infants in the NICU receive developmental care support from health care providers and families, and clinical staff tracks which activities are experienced by all infants through nursing documentation. Clinical staff uses a standard care path to guide which types of activities are appropriate for an infant depending on the maturity and health status. Families are generally encouraged to talk, read, and sing to their infants when visiting, if the infant is deemed in the appropriate stage. Auditory setup will be the same for neonates in the C group; however, these neonates will not hear speech recordings. This procedure ensures that parents remain blinded to the group randomization.

#### Participant retention plan

To maintain contact in preparation for longitudinal assessments of language and brain development outcomes, we plan to contact the primary caregiver of the infant participant at 6 months AA in concert with routine 6-month AA high-risk infant follow-up clinic visitation or via phone call, text message, or email for infants who are not followed in the clinical program. We will make sure that the contact information is up to date and to inform families of study procedures that will be performed for follow-up visits.

### Outcome assessment methods

The primary outcome is language development (domain) assessed through the MacArthur-Bates CDI (measure) at (metric) 18 months AA (time point), quantified as expressive and receptive language raw scores and aggregated as group means (aggregation). The secondary outcome is white matter development (domain) assessed through dMRI scans (measure) collected at (metric) two different time points (time point): (1) prior to hospital discharge (neonatal time point) and (2) at 12 months AA (1-year time point), quantified with the dMRI metric (MD) and aggregated as group means (aggregation).

### Primary outcome: language development

#### MacArthur-Bates CDI

In order to measure language development and production, parents of infant participants will complete the CDI: Words and Gestures Questionnaire [46] at 12 months AA and 18 months AA via web form or mail form with guidance from a trained clinical research assistant. The CDI Words and Gestures Questionnaire is a parent checklist that evaluates both verbal and non-verbal communication, as well as some aspects of play. Split into two parts, “The Early Words” section examines infants’ intentional linguistic communication, understanding, vocabulary, and language-based social interactions, while the “Actions and Gestures” section gauges communicative gestures, play, and symbolic understanding. As these metrics of language development are related to measures of processing speed and predictive of later language outcomes, the CDI Words and Gestures Questionnaire will serve as our primary measure of language development.

### Secondary outcomes: white matter development

#### Scanning procedures

At each age point, dMRI scans will be collected on a 3-T MRI (GE-Discovery MR750) using multi-slice scanning. The MRI scanning duration is approximately 30 min. Sequence parameters are optimized for neonates and infants and are constant across age points. Neonatal scans will be performed at LPCH as part of the routine medical care prior to hospital discharge. Infant scans will be collected at the Center for Cognitive and Neurobiological Imaging at Stanford. We follow established procedures to ensure safety and successful scan acquisition (e.g., scanning at bedtime, noise-canceling headphones, swaddling to reduce movement) [47].

#### Diffusion MRI parameters

We will collect two dMRI scans that vary in terms of b-values (700 s/mm^2^ and 1500 s/mm^2^). Each scan is collected at 2.0 mm^3^ spatial resolution with full-brain coverage and 60 non-collinear directions. Six volumes are acquired at b = 0. We employ a multi-slice echo-planar imaging (EPI) protocol to ensure rapid image acquisition (~ 3 min). To correct for EPI distortions, we collect an additional short scan with 6 non-diffusion-weighted volumes with reversed phase encoding (posterior-anterior). The rationale for a low b-value (b = 700) is to optimize the signal-to-noise ratio for measuring diffusion in the underdeveloped neonate brain. We will also collect a high-resolution T1-weighted scan for anatomical reference. We use a 3D fast spoiled gradient sequence.

#### Neuroimaging pre-processing and tractography

We plan to use established pipelines for image pre-processing and tractography. These pipelines rely on a combination of open-source software, including mrDiffusion [48], Statistical Parametric Mapping [49], FMRIB Software Library [50], mrTrix3 [51], Advanced Normalization Tools [52], and Automated Fiber Quantification [53]. Pre-processing includes the alignment to T1-weighted anatomical scan, de-noising, and corrections for participant motion, eddy currents and EPI distortions, and model fitting to obtain the three eigenvalues (λ1, λ2, λ3) used to compute MD, FA, radial diffusivity, and axial diffusivity. Tractography procedures include steps for producing the whole-brain tractogram, segmentation of individual white matter fiber groups in native space, and estimation of diffusivity metrics within specified tract segments [53].

#### Control variables and covariates

To characterize the sample and ensure balance across groups in analyses for treatment effects, we will prospectively collect the following information from electronic medical records and parent report questionnaires to control for (1) major medical complications associated with prematurity, including GA, birth weight, sex, history of antenatal steroid usage, infection, number of days of intubation and oxygen, and X-ray changes consistent with chronic lung disease; (2) PMA at MRI scan; (3) the number of nights of exposure to study treatment; and (4) sociodemographic factors associated with later language outcomes, including the SES of primary caretakers as measured by the HI [43], the frequency and number of languages spoken by adult and child household members, and number of siblings.

We will also obtain metrics to ensure balance across groups on the basis of the amount of language exposure experienced by infants in their home environments. To collect data on naturalistic maternal/caregiver input during the infancy period, we plan to use the Language ENvironment Analysis (LENA) recording device and software [54]. Families will be asked to perform two full-day (16 h) home LENA voice-recording sessions immediately around the time of the 12 months AA visit for MRI scanning. From this device, we will examine the adult words count measure per hour, normalizing for the length of audio recording, since it is the most accurate measure from LENA at this age when the infants may have limited language production skills [55]. If the groups do not match, we will use this language exposure metric as a covariate in the analyses.

### Analysis plan

#### Power calculations

We calculated the sample size based on power. The significance level is *p* < 0.05 for all analyses. Power estimates are based on the means, standard deviations, and samples size from an RCT neuroimaging study of preterm neonates that showed a significant effect of a parent-based NICU intervention on diffusion metrics measured at close-to-term age (Cohen’s *d* = 1.03) and developmental outcomes (Cohen’s *d* = 1.77) measured at 9 months AA [56]. Thus, if we assume an effect size that is more conservative than this earlier study, specifically Cohen’s *d* = 1.0, we will have a power of ß = 0.8, if we enroll a sample size of at least 17 participants per group. Therefore, our planned enrollment of 21 participants per group will yield a power of ß = 0.88 to detect an effect size at that level (Cohen’s *d* = 1.0). If our effect size is even smaller than we anticipate, for example, Cohen’s *d* = 0.9, a planned enrollment of 21 per group will still yield a power greater than 0.8 to detect a treatment effect.

#### Analytic strategy

We anticipate that stratification prior to randomization will result in a close matching of T and C groups on demographic and health variables. Prior to statistical analyses, we will use independent samples t-tests (continuous variables) or chi-square tests (categorical variables) to assess the statistical balance between T and C groups on the basis of GA, sex, SES, clinical complications associated with premature birth, and language exposure during treatment and after hospital discharge. Analyses will use an intention-to-treat strategy [57]. If groups are matched on all variables, we will use independent t-tests (two-tailed) to compare T versus C groups on the primary long-term language outcome and the secondary short-term outcomes that relate to white matter development. Should groups not be balanced, and to account for missing data, we will use linear mixed models: T group as a fixed factor and unmatched or missing variables are random factors. Statistical significance is set at *p* < 0.05. Should an imbalance of twins or multiples occurs, we will perform group analyses including all singletons and one randomly selected twin or multiple from each set [58].

We will perform post hoc univariate and/or non-parametric analyses to determine if neural and clinical outcomes are associated with variations in the dose (i.e., number of hours of maternal speech delivered) or length (i.e., number of nights of delivery) of treatment, controlling for GA and PMA. Post hoc covariance analyses will permit further interpretation of either significant or non-significant treatment effects for short- and long-term neural and language outcomes. We will use multiple regression analyses to predict language outcomes from short-term neural and clinical outcomes, while controlling for group status and measures of language exposure.

### Data collection and management

Data will be de-identified using a participant identification number that is coded independent of group assignment. All data are collected and analyzed without knowledge of the group assignment. Only the principal investigator and research staff will have access to the data. Demographic and clinical data from participants’ electronic medical records will be automatically extracted and entered into a Research Electronic Data Capture (REDCap) database (http://redcap.stanford.edu) that is supported by the Stanford Medicine Research IT team. Parent questionnaires will be completed with paper or electronic REDCap forms whenever possible. The REDCap platform services at Stanford are subsidized by (a) Stanford School of Medicine Research Office and (b) the National Center for Research Resources and the National Center for Advancing Translational Sciences, National Institutes of Health. For neuroimaging data, we transitioned processing to Flywheel (https://flywheel.io/), an integrated imaging processing system that uses cloud-based computing to automate analysis pipelines and allows data archiving and sharing of de-identified data.

### Harms

We do not anticipate adverse events related to exposure to maternal voice recordings. Clinical staff in the nursery already responsible for monitoring the health status of the infants will do so for the entire duration of the intervention period. In the unexpected event that an infant does not tolerate the voice recordings, the intervention will be ended immediately. There is some risk for abnormal findings on MRI scans at 12 months AA. In the event of an abnormal scan, participants will be referred to the Department of Radiology at LPCH (or other institution) for interpretation. The principal investigator assumes responsibility for contacting families about unanticipated abnormal results and will recommend families seek further consultation for their primary care physician when necessary and/or recommended by the consulting radiologist. Any unexpected adverse events will be reported to the Data Safety Monitoring Board and to the Institutional Review Board.

### Ethics

Our RCT was approved by the Stanford School of Medicine Institutional Review Board (IRB). Informed consent is obtained from the infant’s parent/guardian by research staff in accordance with IRB procedures. The intervention will be overseen by a data safety and monitoring committee comprising two neonatologists and a pediatric neuroradiologist at LCPH.

### Limitations of the study

The present intervention may not significantly change long-term language outcomes or white matter development. It is possible that parents who consent to the study may increase their verbal input to infants, thus swamping treatment effects. However, the amount of speech that neonates typically hear is generally limited and will be vastly increased by the proposed intervention. In addition, individual variations in language exposure following discharge from the NICU may swamp out early gains made by the NICU intervention. If we fail to find long-term effects of the intervention, it may be that naturalistic maternal speech overwhelms the effects of the intervention. Such findings would suggest a need for additional language interventions administered within the first postnatal year (e.g., monthly; every 3 months; quarterly) that could be developed and tested as part of future RCTs.

## Discussion

Despite consistent evidence linking preterm birth to delays in language development, few clinical interventions for promoting healthy language development currently exist. To our knowledge, the present RCT is the first designed to assess the impact of increased exposure to maternal speech on long-term language outcomes and white matter brain development. By examining the potential changes induced by increased exposure to maternal speech on white matter connectivity, this RCT may promote changes in infant brain development that may serve as a marker of effective intervention and a precursor of future language development. Therefore, the significance of this RCT resides in that we may be able to prevent neurodevelopmental delays before they manifest and thus move the child toward favorable developmental trajectories, by intervening early in the hospital nursery. The ultimate significance of the proposed research will be to establish the nature and timing of language interventions for improving language and neural outcomes in preterm infants.

## Trial status

Protocol version number and date: NCT04193579, December 10, 2019

Date when recruitment began: November 25, 2019

Approximate date when recruitment will end: December 31, 2021

## Data Availability

De-identified data analyzed as part of the current study will be made available upon request to the corresponding author.

## Abbreviations

AA: Age adjusted for prematurity
CDI: MacArthur-Bates Communicative Developmental Inventories
C group: Control group
dMRI: Diffusion magnetic resonance imaging
FA: Fractional anisotropy
GA: Gestational age
IRB: Institutional Review Board
LPCH: Lucile Packard Children’s Hospital
MD: Mean diffusivity
NICU: Neonatal intensive care unit
PICN: Packard intermediate care nursery
PMA: Postmenstrual age
RCT: Randomized controlled trial
REDCap: Research Electronic Data Capture
SES: Socioeconomic status
T group: Treatment group
